# Endoscopic endonasal approach for loco-regional recurrent clivus chordomas

**DOI:** 10.1016/j.bas.2022.100918

**Published:** 2022-07-30

**Authors:** Matteo Zoli, Federica Guaraldi, Davide Gori, Riccardo Cavicchi, Giacomo Sollini, Sofia Asioli, Marco Faustini-Fustini, Raffaele Agati, Raffaele Lodi, Caterina Tonon, Ernesto Pasquini, Diego Mazzatenta

**Affiliations:** a IRCCS Istituto delle Scienze Neurologiche di Bologna, Programma Neurochirurgia Ipofisi - Pituitary Unit, Bologna, Italy; b Department of Biomedical and Neuromotor Sciences, University of Bologna, Italy; c Azienda USL di Bologna, Anatomic Pathology Unit, Bellaria Hospital, Bologna, Italy; d Azienda USL di Bologna, ENT Department, Bellaria Hospital, Bologna, Italy; e Programma Neuroradiologia con Tecniche ad Elevata Complessità, IRCCS Istituto delle Scienze Neurologiche di Bologna, Bologna, Italy; f IRCCS Istituto delle Scienze Neurologiche di Bologna, Bologna, Italy; g IRCCS Istituto delle Scienze Neurologiche di Bologna, Programma Neuroimmagini Funzionali e Molecolari, Bologna, Italy

## Abstract

• EEA represents an ideal approach for loco-regional recurrent CCs.
• EEA is well tolerated, with preservation of patients QoL.
• EEA can be considered for patients with perspectives of adjuvant therapies.
• Otherwise, EEA can be considered only in selected cases with a palliative aim.

## 1 Introduction

Among the different skull base neoplasms, clivus chordomas (CCs) are characterized by multiple peculiarities. Indeed, these bone-eroding, infiltrative tumors often present with an aggressive behavior, multiple loco-regional recurrences and, at late stages, also with possible metastases to target organs (Frezza et al., 2019; Stacchiotti et al., 2015; Cavallo et al., 2020). According to large clinical series, more than 50% of the patients with a CC develop one or more loco-regional recurrences, even after gross tumor resection (GTR) and adjuvant radiotherapy (Frezza et al., 2019; Stacchiotti et al., 2015; Cavallo et al., 2020; Yaniv et al., 2020; Snyderman and Gardner, 2020; Kim et al., 2018; Fernandes Cabral et al., 2018). Usually, recurrences occur after 5–10 years from the initial treatment, and represent a challenge for all the specialists involved in the management of these patients (i.e. neurosurgeons, radiation/medical oncologist and specialists in palliative cares) (Frezza et al., 2019; Stacchiotti et al., 2015; Cavallo et al., 2020; Yaniv et al., 2020; Snyderman and Gardner, 2020; Kim et al., 2018; Fernandes Cabral et al., 2018). Surgical and adjuvant treatments of CCs have significantly improved in last years (Frezza et al., 2019; Stacchiotti et al., 2015; Cavallo et al., 2020). In particular, the endoscopic endonasal approach (EEA) has been widely adopted by most of worldwide centers to treat of the majority of CCs (Labidi et al., 2016; Cavallo et al., 2020; Fernandez-Miranda et al., 2014; Zoli et al., 2018). EEA exploits a natural corridor, given by the nasal and paranasal sinuses, that allows to directly approach these tumors, along their same axis of growth, achieving the largest possible exeresis of the neoplasm, while reducing surgical morbidity by avoiding any manipulation of neural and vascular structures (Labidi et al., 2016; Cavallo et al., 2020; Fernandez-Miranda et al., 2014; Zoli et al., 2018). Targeted radiation treatments with photons/heavy particles have also been implemented to improve the control on disease recurrences, with an overall acceptable safety profile (Cavallo et al., 2020; Fernandez-Miranda et al., 2014; Zoli et al., 2018; Labidi et al., 2016; Evans et al., 2020; Kim et al., 2017; Heery, 2016). Finally, few experimental drugs, as imatinib and sorafenib, seem efficacious in blocking the tumor progression in patients with advanced stages of the disease (Colia and Stacchiotti, 2017; Stacchiotti et al., 2018; Tamborini et al., 2010). Thanks to active translational research in this field, it is expected that other medical options could be available soon (Heery, 2016; Colia and Stacchiotti, 2017; Marucci et al., 2014).

Similarly to primitive tumors, loco-regional recurrences of CCs can be treated with all these options, too (Frezza et al., 2019; Stacchiotti et al., 2015; Cavallo et al., 2020). However, the achievement of local control is often more challenging and, although all efforts should be taken to reach this result, the role of surgical re-resection in these cases is still debated (Frezza et al., 2019; Stacchiotti et al., 2015; Cavallo et al., 2020). A recent position statement suggested to consider surgery in tumors assessed amenable to gross tumor resection, or in very selected cases with a cytoreductive palliative aim, in both cases eventually adjusted by radiation or chemo/radiation treatments, depending on tumor and patient characteristics (Stacchiotti et al., 2017). However, no consensus has been reached on the most appropriate surgical approach for these cases, while clinical outcomes of surgical re-treatment remain uncertain (Stacchiotti et al., 2017).

This study aimed at evaluating the role of EEA for loco-regional recurrent CCs, assessing its the surgical results and the patients clinical outcome, including the overall survival (OS) and progression free survival (PFS), and determining the prognostic factors of further local relapses.

## 2 Materials and methods

All consecutive patients with CCs, operated at our Center since 1998 (year of introduction of EEA at our Institution) to June 2021, were retrospectively revised to identify those operated for loco-regional recurrences. Patients previously operated at our or at different centers, who developed a surgical loco-regional recurrence at follow-up, have been enrolled. Other inclusion criteria were: 1. histological confirmation of chordoma; 2. treatment via EEA; and 3. availability of clinical and radiological data at diagnosis and follow-up. Patients naïve for surgery and undergoing EEA to treat the primary tumor, were used as controls. Those cases who had previously undergone only biopsy, or who had been treated with sole radiation therapy, were considered as primary resection. Cases with unavailable complete medical records were excluded. No patient was lost at follow-up.

### 2.1 Pre-operative management

Our protocol for patient clinical and surgical management has been previously reported (Zoli et al., 2018). Briefly, all patients underwent a multidisciplinary pre-operative evaluation that included the collection of past medical history, with special attention to previous surgical or radiation therapies for the CC, a neurological evaluation, and the assessment of Karnofski performance score (Zoli et al., 2018). In case of tumor extension to the sellar region, patients also underwent biochemical evaluation of the hypothalamic-pituitary function (basal in all patients, stimulated whenever required) and an endocrinological consultation, while, whenever the opto-chiasmatic cistern was involved, patients underwent an ophthalmological examination with the assessment of visual acuity and visual field. Preoperative neuroradiological assessment consisted of a brain MRI with gadolinium contrast medium, a brain CT with angiographic sequences, and a body CT-scan to exclude metastases. The pre-operative MRI and CT-scan was used to define the tumor localization in the clivus (upper, middle or lower clivus), and the presence of intra-dural extension.

### 2.2 Surgical Technique

Surgery was performed under general anesthesia with oro-tracheal intubation. The patient was placed in a semi-sitting position with the thorax slightly elevated (20°). Laryngopharynx was packed with gauzes to prevent blood leakage in the upper respiratory tract. Straight and angled (30°) endoscopes with HD cameras (SPIES, Karl Storz, Tubingen, Germany) were used, together with neuronavigation (S8 MEDTRONIC, Louisville, CO. USA), based on CT-angiograms (CTAs) and processed through StealthMerge Software (MEDTRONIC, Louisville, CO. USA).

**Fig. 1:**
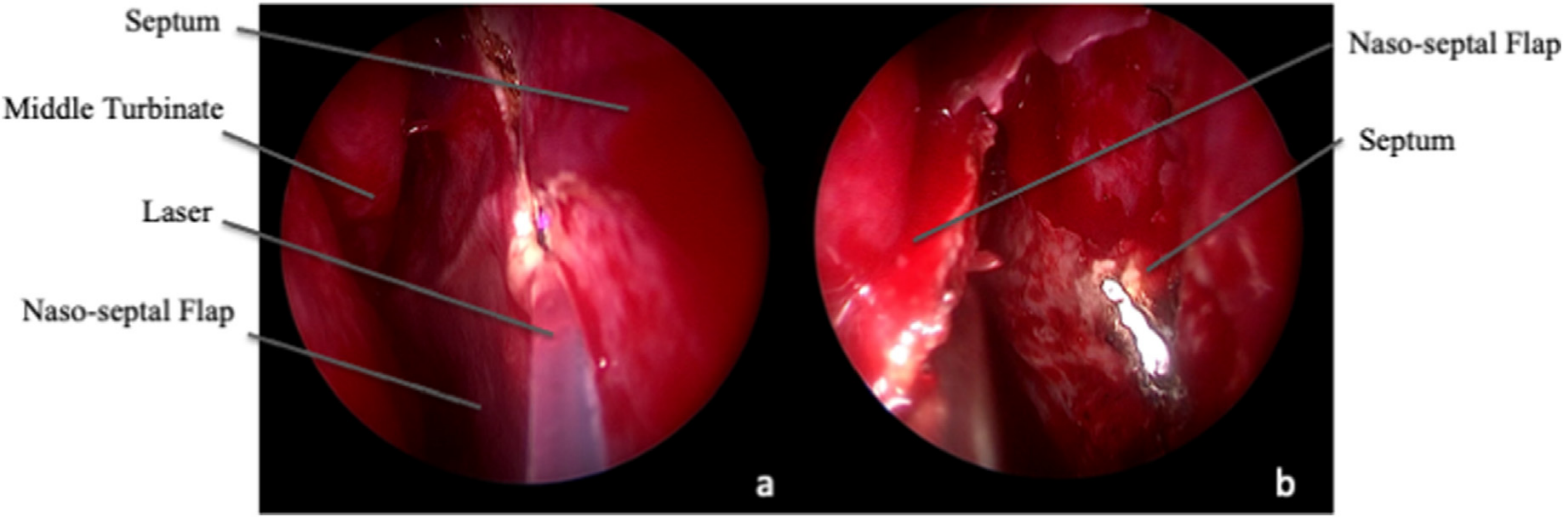
Intra-operative endoscopic image, 0° scope. Approach phase. A, B and C It is important to harvest the naso-septal flap as soon as possible, especially in patients already operated via EEA, to avoid any damage to potentially relevant homologous materials for the closure at the end of surgery. In this case, the mucoperiustium and its pedicle at the right side of the septum were kept intact from previous surgery and this flap still was available. D. After its preparation, the flap can be located in the rhinopharynx or in the maxillary sinus, and the previous surgical approach can be enlarged to expose the tumor as larger as possible.

The surgical approach consisted in a transclival corridor, tailored case by case on the tumor location and extension. After harvesting the most appropriate mucoperiosteum flap, surgery started with an anterior large sphenoidomy, and a posterior septostomy. Middle turbinates were usually preserved, and, whenever possible, ethmoidectomy was avoided. After the identification of the vidian canals, the sphenoidal sinus floor was drilled out with a high-speed drill (Midas Rex, MEDTRONIC, Louisville, CO. USA), then the clivus was exposed, according to tumor extension at the pre-operative neuroimaging (Fig. 1). Based on CC location, the approach was expanded by combining a transpterygoid route for the lesions invading the cavernous sinus, or a transmaxillary corridor for tumors extending toward the Meckel cave or the pterygopalatine and infratemporal fossae.

**Fig. 2:**
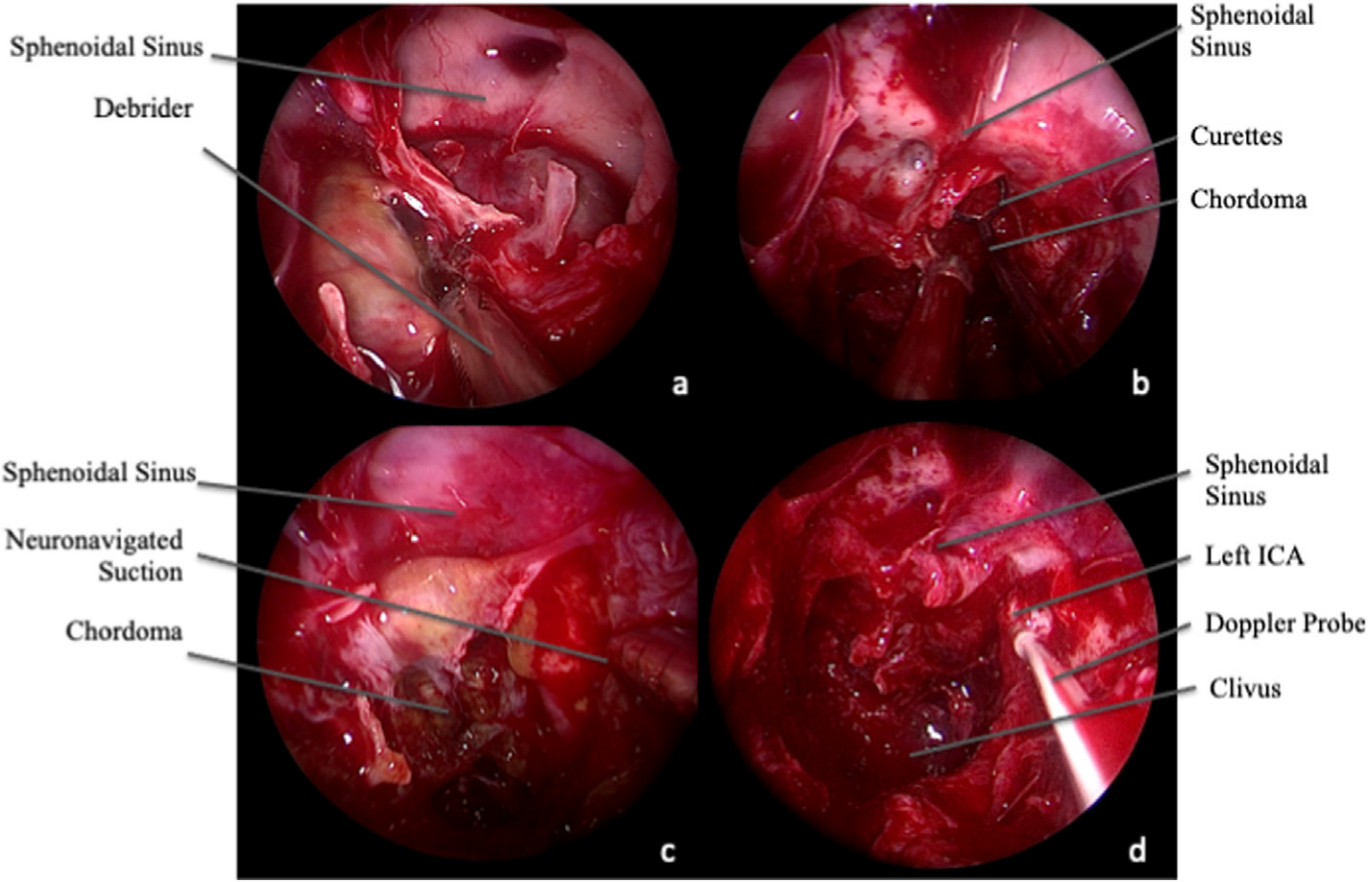
Intra-operative endoscopic image, 0° scope. Tumor removal. AA. The extracranial portion of the tumor can be resected with the debrider, which is particularly effective also in presence of scars from previous surgeries. B. In its intracranial portion, it is preferable to remove the tumor with curretes and suction, to reduce the risk of surgical complication. Ultrasonic aspirator can be also used, especially for firm CC. C and D. During tumor removal, it is necessary to check the location of the carotid arteries by neuronavigation (C) and intra-operative Doppler (D), reducing the risk of injuries to these vessels.

**Fig. 3:**
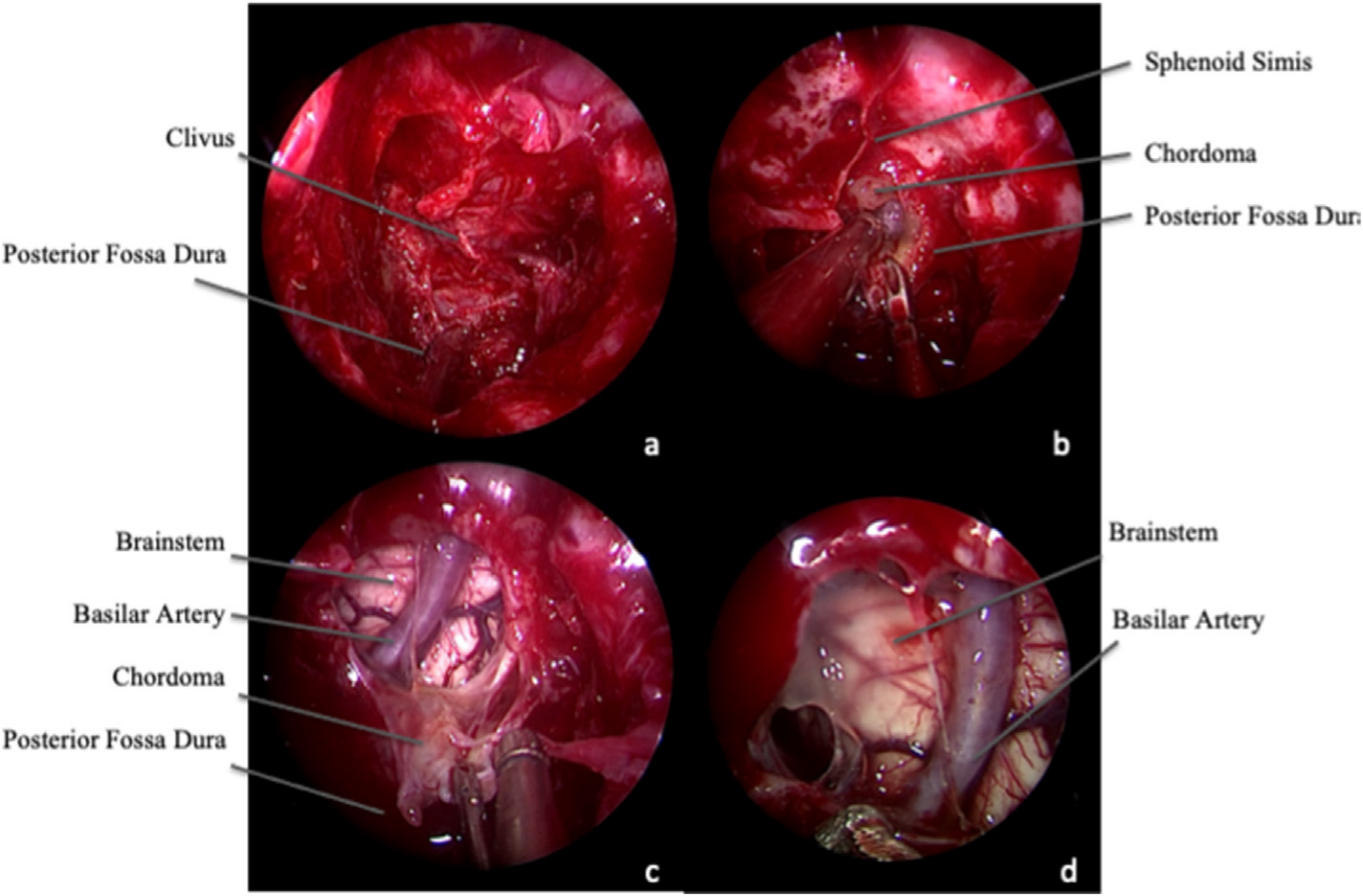
Intra-operative endoscopic image, 0° and 30° scope. Tumor removal. A. After resection of the extradural portion of tumor, the CC can be followed in the intradural space. B. The dura is cut, and the tumor progressively mobilized. C and D. Inspection of the surgical field at the end of surgery both with 0° (C) and 30° (D) scope, to detect any possible tumor remnants.

Tumor resection was performed with a four hands technique, after fixing the endoscope on a mechanical holder. CC were initially debulked in the central portion with curettes, then the tumor was followed to achieve GTR, whenever possible (Fig. 2, Fig. 3).

**Fig. 4:**
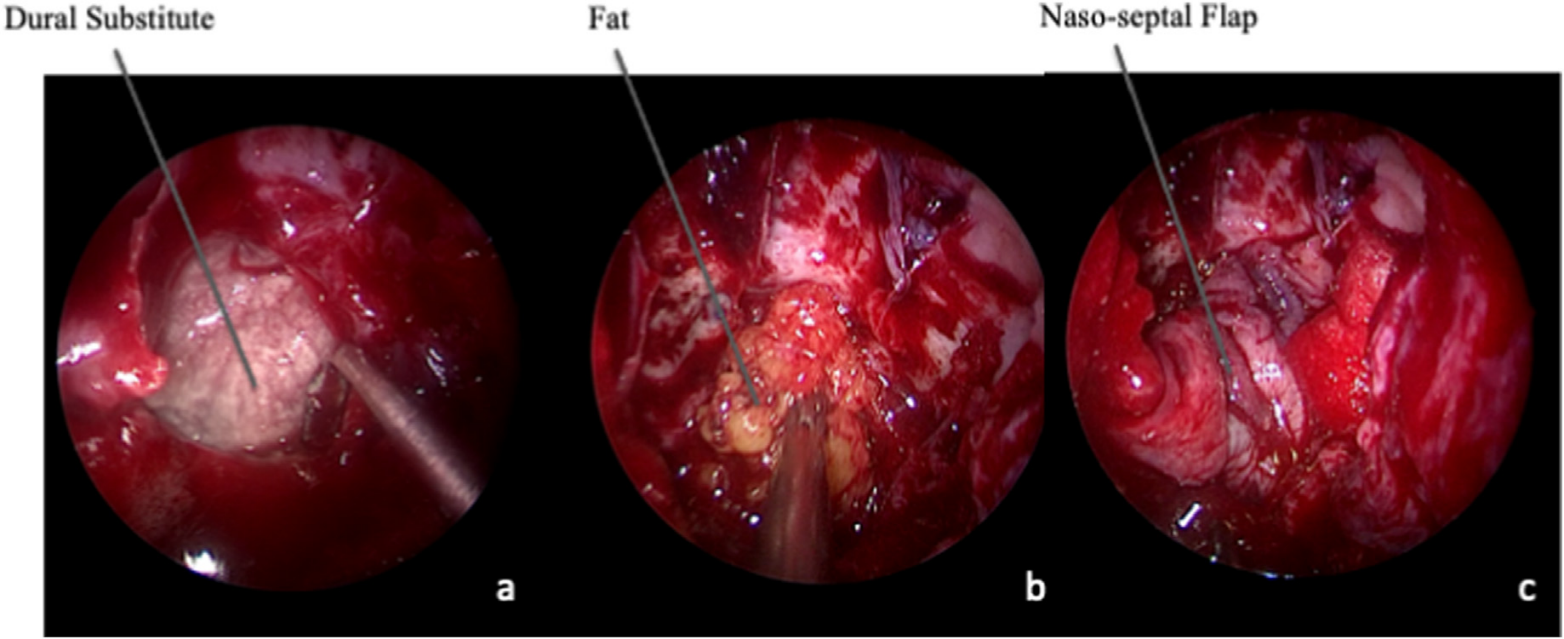
Intra-operative endoscopic image, 0° scope. Closure. If no CSF leak was observed, the surgical cavity is filled with fat and eventually covered with mucoperiosteum, especially if carotid arteries have been exposed to avoid delayed ruptures. A. In case of CSF leak, closure includes an intradural layer with a heterologous dural substitute. B. Afterwards, fat is placed to fill the cavity. C. The closure is covered with the mucoperiosteum flap, harvested at the beginning of surgery. Whether no pedicled flaps is available, it should be considered the use of alternative techniques, as galea or temporalis fascia flap, depending on tumor extension.

Whether an intra-operative CSF leak was observed, a watertight plastic repair was performed. Along the years, our closure technique has implemented. Nowadays, we prefer a multilayer technique with dural substitute, abdominal fat, eventually bone or cartilage and a pedicled flap obtained from the septum or by rhinopharynx for lower clivus tumors (Fig. 4). Finally, sphenoid sinus was packed with gelfoam, and a single Merocel (Merocel Corp., Mystic, CT) was inserted in both nostrils.

### 2.3 Post-operative management

**Fig. 5:**
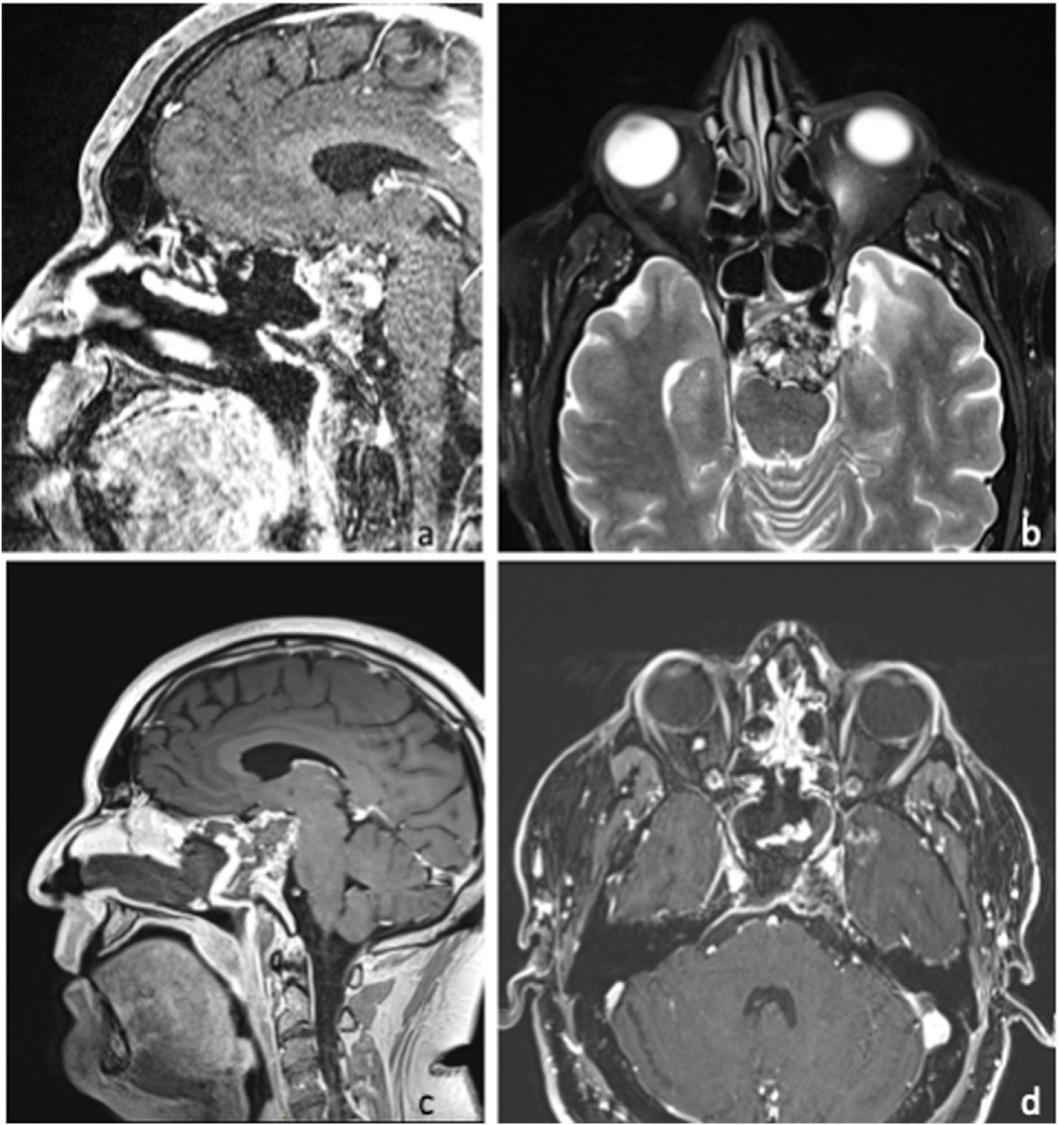
MRI, an exemplificative case of loco-regional recurrent chordoma, underwent surgery with a resective aim is reported. The patient of 32 years old was already treated by an EEA, followed by heavy particles radiotherapy. After 42 months, he presented with loco-regional recurrence, located in the middle/inferior third of the clivus with extra- and intra-dural extension, causing diplopia for CN VI palsy. A and B. T1-w with gadolium, sagittal and coronal view, showing the recurrent tumor. An EEA was planned with the aim to remove the tumor, followed by a second radiation therapy. C and D. T1-w with gadolium, sagittal and coronal view, demonstrating the complete tumor removal. Diplopia regressed and the patient is still alive at follow-up of 24 months, without any further tumor progression.

**Fig. 6:**
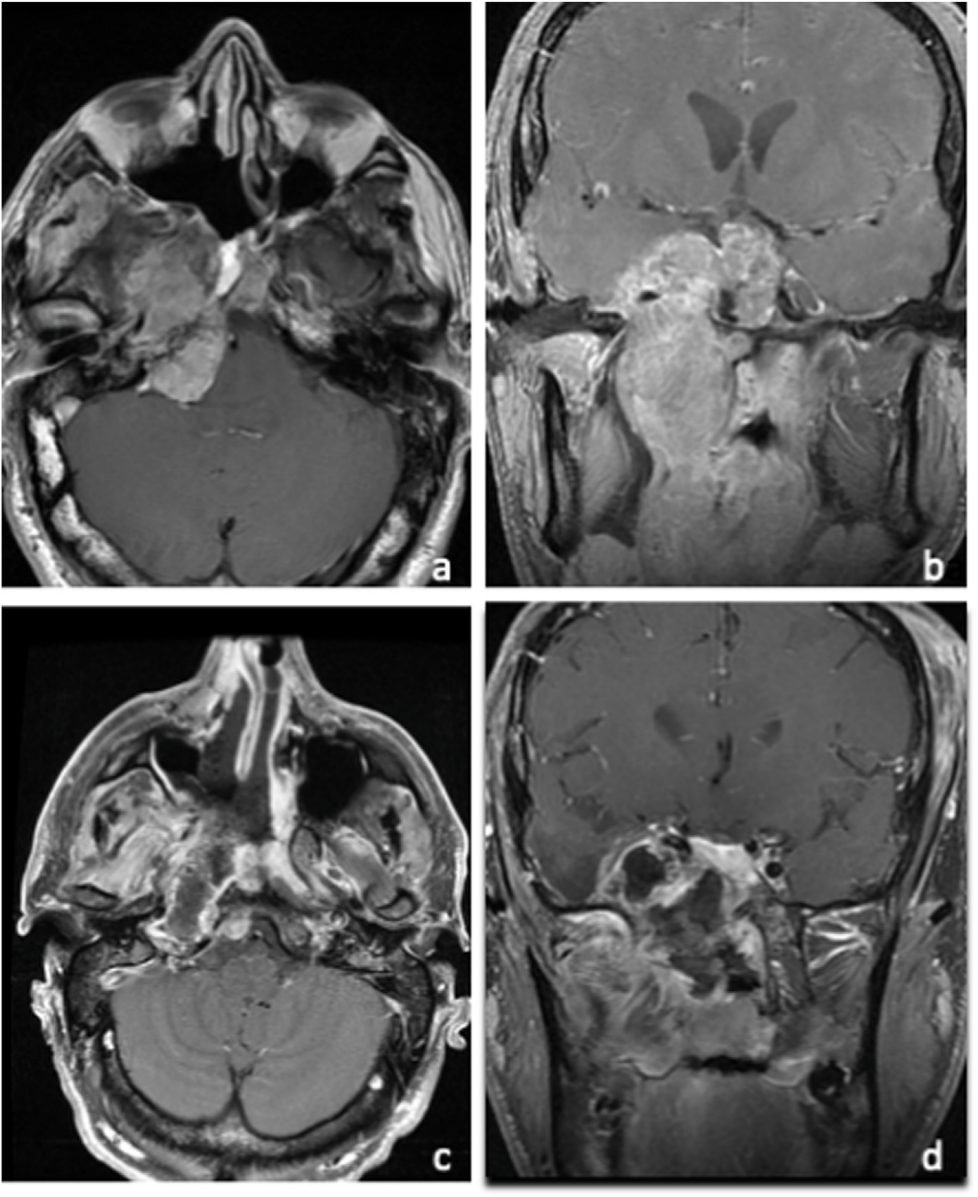
MRI, an exemplificative case of loco-regional recurrent chordoma, underwent surgery with a palliative aim is reported. The patient of 62 years old was already treated by one transcranial approach and one EEA, followed by one radiosurgery and heavy particles radiotherapy. After 24 months, he presented with a progression of the previous remnant, located in the middle/inferior third of the clivus with extra- and intra-dural extension, causing nasal obstruction, trigeminal pain resistant to medications and diplopia. A and B. T1-w with gadolium, sagittal and coronal view, showing the recurrent tumor. An EEA was planned with the aim to decompress the neural structures to alleviate the patients symptoms. C and D. T1-w with gadolium, sagittal and coronal view, demonstrating the partial tumor resection. At follow-up, the trigeminal nevralgia was controlled by medical therapy and diplopia and nasal obstruction regressed. The patient was addressed to the palliation care unit, and he died 18 months later.

Surgical complications were retrieved from medical records. Surgical intent was considered as resective (if the largest possible surgical resection was planned), or palliative (if it aimed at a citoreductive debulking), based on the information collected by the report of the pre-operative multidisciplinary board discussion of each case (Fig. 5 and 6). The definitive diagnosis was based on the histological examination, that was blindly confirmed by two expert pathologists (in case of discordance, cases were discussed to find an agreement). All patients were extubated after surgery, and oral feeding was restored on the same day. All patients underwent brain MRI within 72 ​h after surgery.

Neurological examination, MRI with gadolinium contrast medium, and endocrinological/ophthalmological evaluation and tests (according to previously mentioned criteria) were repeated 3 months after surgery, then every 3, 6 and 12 months. Body CT-scans were repeated at regular intervals. Adjuvant therapy (photon/proton radiotherapy, or other radiation/chemotherapy) was discussed in each single case by the multidisciplinary team during the follow-up. Patient quality of life (QoL) was analyzed by the results of Katz index of independence in the activities of daily living, obtained by self-report or family/care-giver report if the patient was unable to respond autonomously (Table 1) (Katz et al., 1963). Peri-operative mortality was defined as death occurring within 30 days from surgery. Mean follow-up was of 48 ​± ​36 months.

**Table 1:** Criteria for the definition of the level of functional patient outcome (adapted from Katz et al., *JAMA*, 1963) (Chen et al., 2022).

Level	Functional Outcome
**1**	complete autonomy in daily activities and social and at work/scholar tasks
**2**	partial autonomy in daily activities and social and work/scholar tasks
**3**	occasional external support necessary for daily life and impossibility to fulfill any social and work/scholar tasks, i.e. a semi-dependence condition
**4**	Daily life absolutely dependent from continuous external support, i.e. a condition of absolute dependence

The extent of tumor resection was assessed at the MRI performed at 3-month follow-up and was defined as gross total (GTR) if no tumor remnant was visible, subtotal (STR) if the residual tumor was less than 20% of the original mass, and partial (PTR) if the tumor remnant was greater than 20% of the original neoplasm. Loco-regional recurrences were defined as tumor re-growth in the clival region identified at follow-up MRIs, while intra- or extra-central nervous system metastases were identified by body CT-scan. Clinical follow-up evaluations were used to assess patient outcome. Progression free survival (PFS) was defined as the time passed between surgery and the occurrence of a local recurrence, while overall survival (OS) as the time passed between surgery and patient death.

## 3 Statistical analysis

The primary study outcome was to analyze the OS and the PFS of locally relapsed CCs treated by EEA with respect to primary surgeries. Secondary outcomes were the assessment of the resection rate, surgical morbidity, and clinical outcome in these patients.

Using a backward-stepwise approach analysis, age, sex, KPS at surgery, pre-operative symptoms, type and timing of previous treatments, tumor size and location in the clivus, intra-/extra-dural extension, resection rate and histological features were weighted as predictors of local relapse for use in the final multiple logistic regression analysis. A multiple logistic regression was performed using the principles of parsimony and biological plausibility. The most important determinants were used to build Kaplan-Meier survival curves for the definition of PFS in patients treated for primary lesions and local recurrence. Then, a subgroup analysis based on the aim of surgery (curative vs. palliative) was performed in the group of patients treated for CC recurrence.

Continuous variables were expressed as mean ​± ​SD, while categorical variables as absolute (n) and relative frequency (%). A p-value < 0.05 was considered statistically significant.

Statistical analysis was performed using Stata (StataCorp. 2017. *Stata Statistical Software: Release 15*. College Station, TX: StataCorp LLC).

## 4 Results

The study sample consisted of 54 surgical procedures for loco-regional recurrent CCs. Surgery was planned with a resective aim in 35 (64.8%) cases and palliative in 19 (35.2%). The control group consisted of 69 primary CCs.

## 5 Loco-regional recurrences operated with resective aim

In the 35 patients with local recurrence treated with a resective aim (21 females, 60%; mean age at surgery 55 ​± ​14 yo), the most common neurological presenting symptom was ophthalmoplegia (23, 65.7%), followed by trigeminal neuralgia (2, 5.7%), facial palsy (2, 5.7%) and hemiparesis (2, 5.7%). Five (14.3%) patients presented with altered pituitary function, and 3 (8.7%) with visual disturbances (Table 2). Mean KPS at surgery was 84 ​± ​7. All patients had previously undergone one or more surgeries, followed by radiotherapy in 15 (42.6%) (Table 2). Thirteen tumors (37.1%) were located in the upper/middle clivus, and 13 (37.1%) presented with intradural extension (Table 3). Main histological features are reported in Table 4.

**Table 2:** Clinical features of the series.

		Locally relapses surgeries with resective aim	Locally relapses surgeries with palliative aim	Primary Surgeries
Sex	Females	21 (60.0%)	8 (42.1%)	35 (50.7%)
	Males	14 (40.0%)	11 (57.9%)	34 (49.3%)
Mean Age		55 ​± ​14	54 ​± ​19	51 ​± ​18
Previous Treatment	None	0 (0%)	0 (0%)	69 (100%)
	Transcranial Appr.	11 (31.4%)	6 (31.6%)	0 (0%)
	Transsphenoidal Appr.	27 (77.1%)	18 (94.7%)	0 (0%)
	Radiotherapy	15 (42.6%)	19 (100%)	0 (0%)
Time since first Surgery		127.5 ​± ​349	52 ​± ​71	0 (0%)
Time since first Radiation Treatment		106 ​± ​453	48 ​± ​40	0 (0%)
KPS		84 ​± ​7	75 ​± ​11	87 ​± ​5
Endocrinological Symptoms	None	30 (85.7%)	14 (73.6%)	66 (95.6%)
	Anterior part. Hypopit.	4 (11.4%)	4 (21.1%)	3 (4.4%)
	Anterior compl. Hypopit.	1 (2.9%)	1 (5.3%)	0 (0%)
	DI	0 (0%)	0 (0%)	0 (0%)
	Panhypopit. with DI	0 (0%)	0 (0%)	0 (0%)
Visual Acuity Deficit	Yes	3 (8.7%)	5 (26.3%)	5 (7.3%)
	No	32 (91.3%)	14 (73.7%)	64 (92.7%)
Visual Field Deficit	None	32 (91.3%)	12 (63.1%)	59 (85.5%)
	Quadrantopia (1 or less quadrant)	1 (2.9%)	0 (0%)	0 (0%)
	Incomplete Bitemporal Hemianopia	0 (0%)	4 (21.1%)	7 (10.1%)
	Complete Bitemporal Hemianopia	1 (2.9%)	1 (5.3%)	3 (4.4%)
	Quadrantopia (more than 2 quadrants)	1 (2.9%)	2 (10.5%)	0 (0%)
	Blindness	0 (0%)	0 (0%)	0 (0%)
Neurological Symptoms	None	10 (28.6%)	0 (0%)	25 (36.2%)
	Oculomotion palsy	23 (65.7%)	18 (94.7%)	35 (50.7%)
	Trigeminal Nevralgia	2 (5.7%)	9 (47.4%)	0 (0%)
	Disphagia/disphonia	0 (0%)	2 (10.5%)	1 (1.4%)
	Facial palsy	2 (5.7%)	1 (5.3%)	1 (1.4%)
	Hemiparesis	2 (5.7%)	3 (15.8%)	2 (2.9%)
	Intracranial hypertension	0 (0%)	0 (0%)	1 (1.4%)
	CN XII palsy	0 (0%)	1 (5.3%)	4 (5.8%)

Legend: KPS: Karnofski performance score; Appr.: approach; Part. Hypopit.: partial hypopituitarism; Compl. Hypopit.: complete hypopituitarism; DI: diabetes insipidus; CN: cranial nerve.

**Table 3:** Neuroradiological features of the series.

		Locally relapses surgeries with resective aim	Locally relapses surgeries with palliative aim	Primary Surgeries
Size	<3 ​cm	11 (31.4%)	2 (10.5%)	(26.1%)
	>3 ​cm	24 (68.6%)	17 (89.5%)	51 (73.9%)
Location	Upper clivus	5 (14.4%)	3 (15.8%)	4 (5.8%)
	Upper/middle Clivus	13 (37.1%)	6 (31.5%)	27 (39.1%)
	Middle Clivus	4 (11.4%)	3 (15.8%)	8 (11.6%)
	Middle/Lower Clivus	3 (8.6%)	3 (15.8%)	6 (8.7%)
	Lower Clivus	4 (11.4%)	0 (0%)	6 (8.7%)
	Holoclival	6 (17.1%)	4 (21.1%)	18 (26.1%)
Dural Extension	Extradural	10 (28.6%)	2 (10.5%)	13 (18.9%)
	Dural Infiltration	12 (34.3%)	9 (47.4%)	30 (43.4%)
	Partially Intradural	13 (37.1%)	8 (42.1%)	22 (31.9%)
	Complete Intradural	0 (0%)	0 (0%)	4 (5.8%)

**Table 4:** Surgical Results of the series.

		Locally relapses surgeries with resective aim	Locally relapses surgeries with palliative aim	Primary Surgeries
Histology	chondroid c.	1 (2.9%)	0 (0%)	7 (10.1%)
	conventional c.	32 (91.4%)	16 (84.3%)	61 (88.5%)
	dedifferentiated c.	2 (5.7%)	3 (15.7%)	1 (1.4%)
Tumor Resection	GTR	18 (51.4%)	6 (31.5%)	50 (72.5%)
	STR	16 (45.7%)	9 (47.4%)	17 (24.6%)
	PTR	1 (2.9%)	4 (21.1%)	2 (2.9%)
Morbidity	CSF leak	2 (5.7%)	0 (0%)	3 (4.3%)
	Haemorrage	0 (0%)	1 (5.3%)	1 (1.4%)
	Ischemia	0 (0%)	0 (0%)	0 (0%)
	Epistaxis	0 (0%)	0 (0%)	0 (0%)
	Carotid Rupture	0 (0%)	0 (0%)	2 (2.9%)
	III CN palsy	0 (0%)	0 (0%)	0 (0%)
	VI CN palsy	0 (0%)	1 (5.3%)	7 (10.1%)
Adjuvant Treatment	Radiation Tr.	22 (62.9%)	7 (36.8%)	65 (94.2%)
	Chemo Tr.	2 (5.7%)	2 (10.5%)	0 (0%)
	Radio and chemo Tr.	1 (2.9%)	0 (0%)	0 (0%)
	Palliative Care	0 (0%)	5 (26.3%)	0 (0%)

Legend: chondroid c.: chondroid chordomas; conventional c.: conventional chordomas; dedifferentiated c.: dedifferentiated chordomas; GTR: gross tumor removal; STR: subtotal tumor removal, PTR: partial tumor removal; CN: cranial nerve, Tr.: treatment.

GTR was achieved in 18 patients (51.4%) (Table 4). Surgical complications consisted of 2 CSF leaks (5.7%), that were promptly re-operated, to prevent meningitis. No perioperative mortality was observed. After surgery, ophthalmoplegia normalized or improved in 11 (47.8%) cases, and trigeminal neuralgia in 1 (50%); the other patients with pre-operative neurological deficit remained unchanged and no additional neurological deficits were observed (Table 5). Post-operative visual worsening was observed in one patient with an upper third chordoma with close relationship with the optic chiasm, probably due to its surgical manipulation during tumor removal. The same patient also developed post-operative central hypocortisolism.

**Table 5:** Symptoms outcome.

		Locally relapses surgeries with resective aim			Locally relapses surgeries with palliative aim			Primary Surgeries		
		norm/impr	unch.	wors.	norm/impr	unch.	wors.	norm/impr	unch.	wors.
Endocrinological Symptoms	None	0	26	4	0	14	0	0	64	2
	Anterior part. Hypopit.	0	4	0	0	4	0	0	3	0
	Anterior compl. Hypopit.	0	0	1	0	1	0	0	0	0
Visual Acuity Deficit	Yes	0	3	0	0	4	1	3	2	0
	No	0	32	0	0	14	0	0	64	0
Visual Field Deficit	None	0	31	1	0	12	0	0	59	0
	Quadrantopia (1 or less quadrant)	0	1	0	0	0	0	0	0	0
	Incomplete Bitemporal Hemianopia	0	0	0	0	4	0	5	2	0
	Complete Bitemporal Hemianopia	0	1	0	0	0	1	1	2	0
	Quadrantopia (more than 2 quadrants)	0	1	0	0	2	0	0	0	0
Neurological Symptoms	None	0	10	0	0	0	0	0	20	5
	Ocular palsy	11	12	0	6	10	2	25	4	6
	Trigeminal Nevralgia	1	1	0	3	6	0	0	0	0
	Disphagia/disphonia	0	0	0	2	0	0	1	0	0
	Facial palsy	0	2	0	0	1	0	0	1	0
	Hemiparesis	0	2	0	0	3	0	1	1	0
	Intracranial hypertension	0	0	0	0	0	0	1	0	0
	CN XII palsy	0	0	0	1	0	0	2	2	0

Legends: Part. Hypopit.: partial hypopituitarism; Compl. Hypopit.: complete hypopituitarism; DI: diabetes insipidus; CN: cranial nerve; Norm.: normalized; Impr.: improved; Unch.:unchanged; Wors.: worsened.

**Fig. 7:**
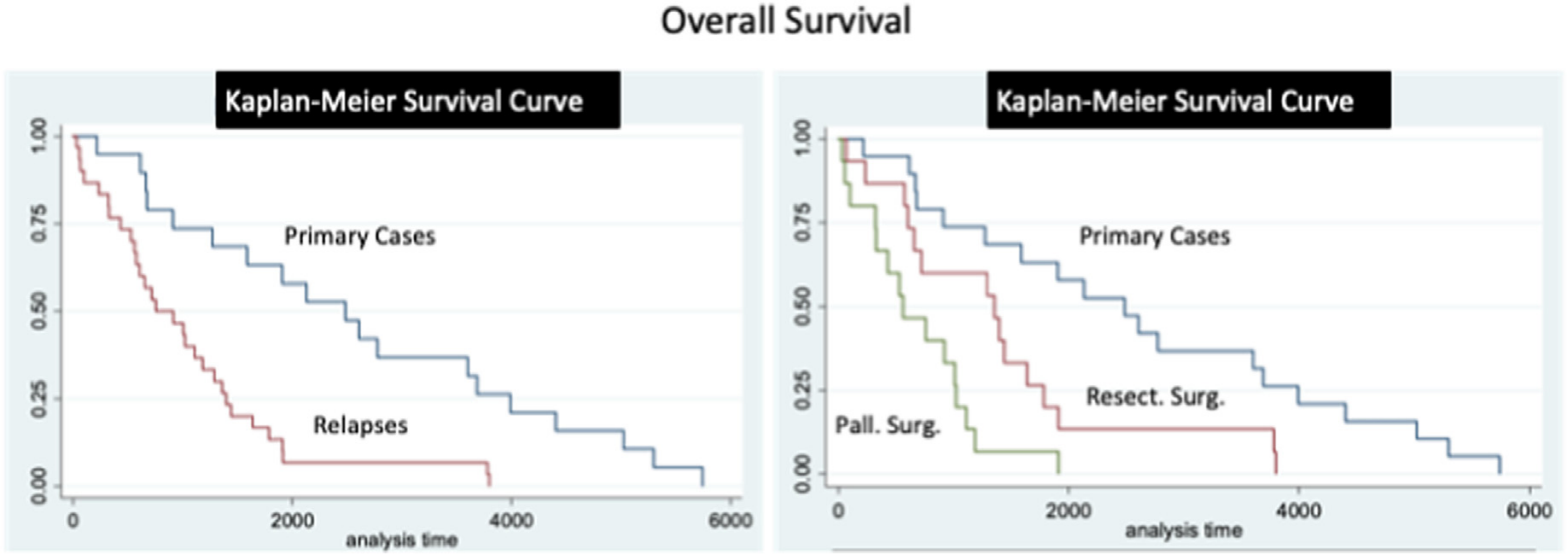
Kaplan-Meier analyses for OS. A. OS for primary and loco-regional recurrent cases. B. OS for primary and loco-regional recurrent cases, considering separately those cases underwent surgery with a resective and palliative aim. Time is expressed in days.

**Fig. 8:**
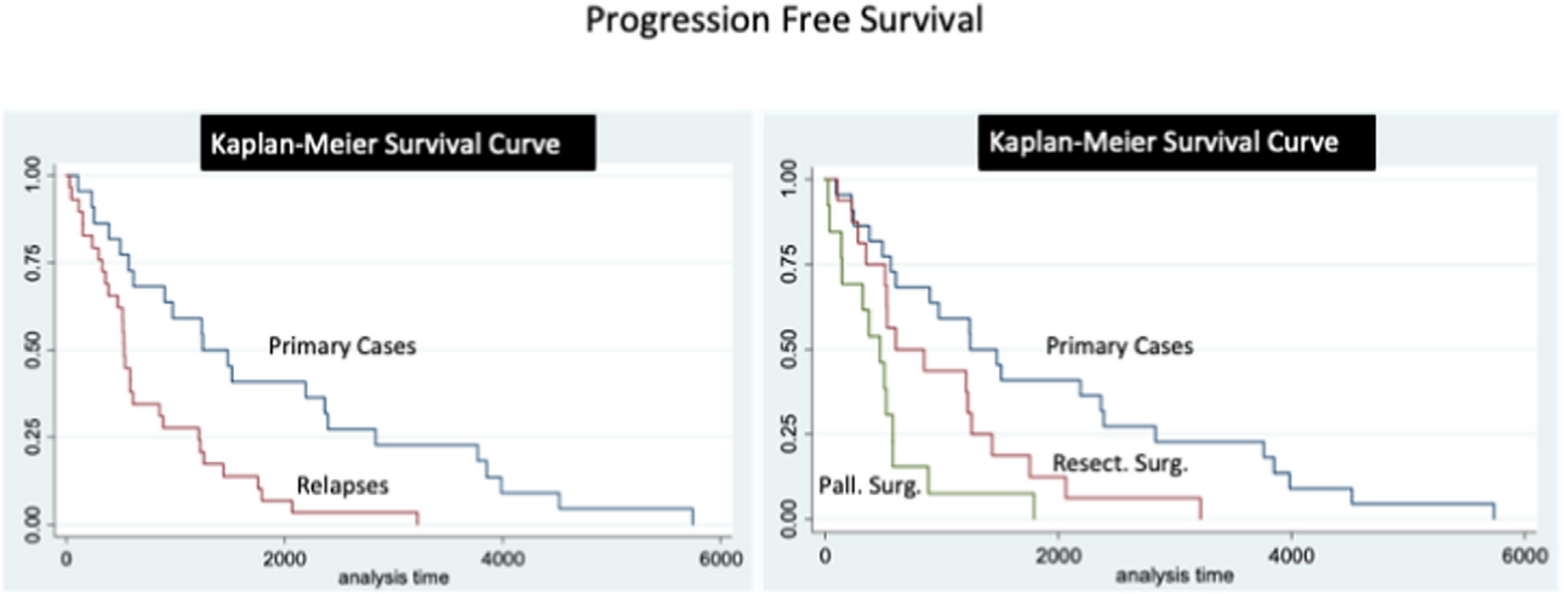
Kaplan-Meier analyses for PFS. A. PFS for primary and loco-regional recurrent cases. B. PFS for primary and loco-regional recurrent cases, considering separately those cases underwent surgery with a resective and palliative aim. Time is expressed in days.

At follow-up, 22 (62.9%) patients underwent radiotherapy with heavy particles (20 were naïve for radiotherapy, while 2 were re-treated) (Table 4). Three patients received chemotherapy, combined with radiotherapy in one case. New loco-regional recurrences were observed in 16 patients (45.7%) after a mean of 33 ​± ​28 months. Fifteen patents (42.9%) deceases for tumor progression after a mean follow-up of 47 ​± ​38 months (Table 6). Post-operative QoL was preserved at pre-operative levels in 18/20 (90%) patients alive at follow-up, and lower in 2 (10%). OS rate was of 95%, 65% and 20% at 1, 3, 5 years, respectively. PFS was of 90%, 50% and 15% at 1,3,5 years (Fig. 7, Fig. 8).

**Table 6:** Adjuvant treatments and patients long-term outcome.

	Local relapses in surgeries with resective aim	Local relapses in surgeries with palliative aim	Primary Surgeries
Recurrence/Progression	16 (45.7%)	14 (73.7%)	26 (37.7%)
Mean Time after Recurrence (in months)	33 ​± ​28	16 ​± ​15	61 ​± ​53
Death	15 (42.9%)	14 (73.7%)	22 (31.9%)
Mean survival Time after Surgery (in months)	47 ​± ​38	21 ​± ​17	82 ​± ​56

## 6 Loco-regional recurrences operated with palliative aim

In the 19 patients treated with a palliative aim (35 females, 50.7%; mean age at surgery 51 ​± ​18 years), then most common symptom was ophthalmoplegia (18, 94.7%), and trigeminal neuralgia 9 (47.4%) (Table 2). Five patients (26.4%) presented with pre-operative hypopitutarism, while 7 (36.9%) with visual disturbances. Mean KPS at surgery was 75 ​± ​11. All patients had been treated with one or more surgeries and radiotherapy (Table 2). Relapse occurred in average 52 ​± ​71 months from surgery, and 48 ​± ​40 months since radiotherapy. Six tumors (31.5%) were located in the upper/middle clivus (31.5%); 8 (42.1%) presented with intra-dural extension (Table 3). At histological examination, 3 cases were de-differentiated (15.7%), while the others were conventional chordomas (Table 4).

GTR was achieved in 6 cases (31.5%) (Table 4). One patient (5.3%) developed a permanent CN VI palsy deficit, and 1 (5.3%) presented with a post-operative hematoma in the surgical field manifesting with reduction of consciousness state in the early post-operative time, requiring a re-intervention via EEA. The patient awakened 48 ​h later and she did not develop any no long-term neurological sequelae. After surgery, ophthalmoplegia normalized or improved in 6 cases (33.3%), and trigeminal neuralgia in 3 (33.3%) (Table 5). Visual deficits worsened in 1 (5.3%) patient, already presenting bitemporal hemianopia. No endocrinological alterations were reported.

At follow-up, 7 (36.8%) patients underwent a second radiotherapy with palliative aim and a salvage chemotherapy was attempted in 2 (10.5%) (Table 4). Five (26.3%) were addressed in the following months to palliative care, while the others were followed-up and addressed to palliative care units at a further stage of the disease. Loco-regional progression was observed in 14 (73.7%) cases after a mean of 16 ​± ​15 months, and 14 (73.7%) patients deceased for tumor progression after a mean of 21 ​± ​17 months (Table 6). QoL was preserved at the pre-operative level in 4 (70%) patients and a lower level in 1 (20%) out of the 5 patients alive at follow-up. OS rate was of 80%, 35%, 10% at 1, 3, 5 years, respectively. PFS was of 75%, 20%, 0% at 1,3,5 years, respectively (Figs. 7 and 8).

## 7 OS and PFS of loco-regional recurrent CCs

OS and PFS of loco-regional relapses were lower than those of primary surgeries (p ​< ​0.001) (Fig. 7, Fig. 8). In particular, OS and PFS were higher in patients treated with a curative aim as compared to those treated for palliation (p ​< ​0.01 and p ​< ​0.01, respectively) (Fig. 7, Fig. 8).

At univariate analysis, GTR appeared a protective factor for local tumor recurrence (p ​= ​0.4), but this association was not confirmed by logistic regression.

**Fig. 9:**
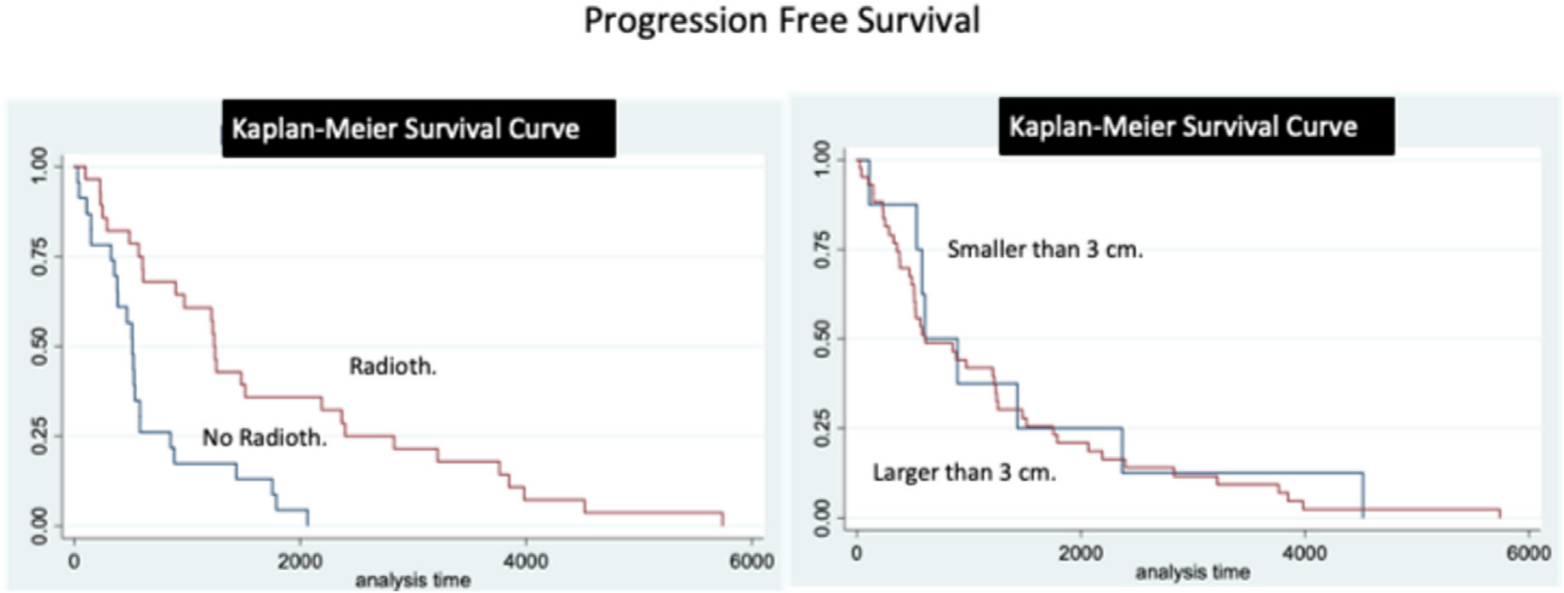
Kaplan-Meier analyses to for the factors influencing the PFS in loco-regional recurrent chordomas. A. PFS for tumors already undergone to radiotherapy or naive for radiotherapy demonstrates a protective effect of radiation therapy. B. PFS for tumors with a size larger or inferior to 3 ​cm. This parameter resulted not associated to a difference in PFS at Kaplan-Meier analysis. Time is expressed in days.

At logistic regression, smaller tumor size and previous radiotherapy were associated with a lower odd of local recurrence (p ​= ​0.05 and p ​= ​009, respectively) (Table 7). At Kaplan-Meier analysis, previous radiotherapy was associated with a higher PFS (Fig. 9).

**Table 7:** Parameters associated to further local recurrences at logistic regression.

	Odds Ratio	Std Err.	z	p	95% Conf. Interv.	
size	2.75	1.4	1.98	0.05	1.01	7.50
**dural ext.**	0.64	0.17	−1.70	0.09	0.39	1.07
**KPS**	0.95	0.26	−1.79	0.07	0.90	1.00
**Rad. th.**	0.30	0.14	−2.63	<0.01	0.12	0.73

Legends: Ext.: extension, KPS: Karnofsky performance status; Rad.: radiation, Th.: therapy.

## 8 Discussion

Our study demonstrated that EEA could have a significant role in the treatment of selected cases of loco-regional recurrent CCs. Indeed, in tertiary referral center as ours, it has permitted to re-operate these tumors with a limited morbidity, mainly consisting of 3.7% rate of post-operative CSF leaks, 1.8% of de novo ophthalmoplegia, and one (1.8%) surgical field hematoma. Moreover, EEA was characterized by an improvement of pre-operative symptoms, particularly of ophthalmoplegia (observed in 41% of cases), trigeminal neuralgia (in 26%), and dysphagia/dysphonia (in both patients with these pre-operative symptoms). Conversely, in our experience, control of long lasting symptoms or either due to tumor infiltration of the neural structures, or to side effects of previous radiation was less effective. Furthermore, differently from primary surgery, no improvement in visual functions was observed in patients treated for local recurrences, and in one case it worsened. The possibilities to alleviate particularly disturbing symptoms (i.e., diplopia, facial pain or swallowing disturbances), with reduced risk of de novo neurological deficits, can lead to the satisfactory QoL referred by the majority of patients at follow-up. Indeed, only 3 (5.5%) reported a re-insertion in the social, familiar and working life at a lower level than pre-operatively, suggesting that EEA can be considered as well tolerated also by patients with recurrent CC.

The role of EEA to control CC progression remains debated, and there is no consensus on the advantages of re-resection of loco-regional recurrences on patients oncological history (Stacchiotti et al., 2017). In our series, GTR was achieved in 40.8% of cases: as expected, tumor resection rate was significantly lower in these patients as compared to those undergoing primary surgery (72.5%). Indeed, the alterations of the normal anatomy andthe presence of scars or fibrosis due to the previous surgeries or radiation therapies represent significant challenges in recurrent CCs surgery (Zoli et al., 2018; Chen et al., 2022; Yousaf et al., 2019). These limitations can be, at least partially, overcome by the use of the neuronavigator and of the intra-operative Doppler, that reduces the risk of surgical mis-orienteering and consequent possible damage of neuro-vascular structures (Chen et al., 2022; Yousaf et al., 2019; Zoli et al., 2018). Indeed, it has been demonstrated that EEA is burdened by a higher risk of internal carotid injury in recurrent CCs, thus the early and accurate identification of these vessels by means of technological devices is of paramount importance (Zoli et al., 2018; Zacharias et al., 2020; Locatelli et al., 2019; Jägersberg et al., 2017; Guinto and Guinto-Nishimura, 2018; Sen et al., 2010; Cannizzaro et al., 2021; OishiTamura et al., 2020; Baldassarre et al., 2021). Similarly, intra-operative electrophysiological monitoring should be used to avoid damage cranial nerves and other neural structures accidental tractions or manipulations (Chen et al., 2022; Yousaf et al., 2019; Kassir et al., 2021; AlQahtani et al., 2020; Zoli et al., 2018).

Interestingly, in our series GTR rate was higher among cases previously operated via EEA than via transcranial approach (46.7% vs. 33%, respectively). This could depend on the fact that the adoption of an intradural corridor by the transcranial approach is associated with a higher risk of adhesions between the tumors and the surrounding neurovascular structures, thus limiting the subsequent chance of resection of local recurrences. Based on our results, we observed that for the vast majority of loco-regional recurrent chordomas, EEA can be considered the approach of choice, which limits are represented by a lateral tumor extension, beyond the cranial nerves and neural structures of the cranial fossae (Zoli et al., 2018; Zacharias et al., 2020; Locatelli et al., 2019; Jägersberg et al., 2017; Guinto and Guinto-Nishimura, 2018; Sen et al., 2010; Baldassarre et al., 2021; Bossi Todeschini et al., 2018). A further limit is the lack of healthy sites for mucoperiostium flap or graft harvesting, for previous radiotherapy or EEA, particularly for those cases at higher risk of intra-operative CSF leak (Zoli et al., 2018; Zacharias et al., 2020; Locatelli et al., 2019; Jägersberg et al., 2017; Guinto and Guinto-Nishimura, 2018; Sen et al., 2010; Baldassarre et al., 2021; Bossi Todeschini et al., 2018). In these patients, alternative closure techniques should be considered, as pedicled galea or temporalis fascia flaps in the EEA pre-operative planning (Zoli et al., 2018; Zacharias et al., 2020; Locatelli et al., 2019; Jägersberg et al., 2017; Guinto and Guinto-Nishimura, 2018; Sen et al., 2010; Baldassarre et al., 2021; Bossi Todeschini et al., 2018). Differently from previous studies, ours failed to demonstrate a significant association between GTR, OS and PFS in loco-regional recurrent chordomas (Zoli et al., 2018; Zacharias et al., 2020; Locatelli et al., 2019; Jägersberg et al., 2017; Guinto and Guinto-Nishimura, 2018; Sen et al., 2010; Baldassarre et al., 2021; Bossi Todeschini et al., 2018). This could be due to the relatively small number of enrolled patients, but, also, to the presence of a higher rate of more aggressive tumors among recurrent CCs than in primary cases, characterized by a higher risk of local relapse, even after the largest possible tumor resection followed by adjuvant radio-/chemotherapies. Unfortunately, for the current lack of effective clinico-radiological or biological markers, which could help to predict a more aggressive tumor behavior, it is not possible to early identify the subgroup of tumors less respondent to therapies and/or at higher risk of local recurrence, distance metastases, and consequently with a worst prognosis. Moreover, our study confirmed the previously reported efficacy of radiotherapy in preventing tumor recurrences, suggesting the limited role of surgery alone in preventing chordoma regrowth (Zoli et al., 2018; Zacharias et al., 2020; Locatelli et al., 2019; Jägersberg et al., 2017; Guinto and Guinto-Nishimura, 2018; Sen et al., 2010; Baldassarre et al., 2021; Bossi Todeschini et al., 2018). Nowadays, the advent of always more modern and sophisticated adjuvant therapies has improved their efficacy in tumor control with limited toxicity (Zoli et al., 2018, Zou et al., 2018; Zacharias et al., 2020; Locatelli et al., 2019; Jägersberg et al., 2017; Guinto and Guinto-Nishimura, 2018; Sen et al., 2010; Baldassarre et al., 2021; Bossi Todeschini et al., 2018). In our series, we have observed that the prognosis of loco-regional recurrent chordomas is poorer than for primary surgeries, but also that patients treated with a resective aim had a significant higher OS and PFS than those treated for palliation. This that can be explained by the higher rate of GTR, but also by the better patient general conditions, allowing subsequent adjuvant treatments, observed in cases operated with a resective aim. Therefore, based on our results, we can confirm the oncological usefulness of second surgery in those patients with loco-regional recurrence of CCs, who can be suitable for a GTR followed by adjuvant radio-/chemotherapies (Frezza et al., 2019; Stacchiotti et al., 2015, 2017; Cavallo et al., 2020; Zoli et al., 2018). Tumor resection via EEA can still be considered with a palliative aim in very selected cases, as, for example, patients with trigeminal neuralgia, facial and orbital pain, nasal obstruction or diplopia to relieve pain and, overall, improve QoL (Stacchiotti et al., 2017). In these situations, it is fundamental to carefully tailor the surgical approach, to limit tumor exeresis to the portion causing pain or other symptoms, while avoiding unjustified surgical aggressiveness, that could further worsen patient QoL because of side effects, without any benefits on prognosis. In the future, the identification of the most active portions of the CC by pre-operative neuroradiological/nuclear medicine techniques could more accurately guide the surgical choice, tailoring the treatment to each case features (Asioli et al., 2020).

Finally, we consider that in order to select those recurrent cases suitable for EEA, the multidisciplinary discussion of each case is of paramount importance not only for a global evaluation of patient conditions and tumor features, but also to plan the most appropriate treatment strategies (i.e. surgery and/or chemo-/radiotherapy, with curative or palliative aim) (Frezza et al., 2019; Stacchiotti et al., 2015; Cavallo et al., 2020). Moreover, surgery for loco-regional recurrent CCs should be performed in dedicated centers, since the results and morbidity of the EEA is strongly dependent on surgeon experience and training for these challenging cases.

Main study limitations are the retrospective design and the relative low number of patients included (although it represents one of the largest series reported in literature), depending on the rarity of CCs. This may have hampered the interpretation of study results not permitting to identify other potential factors with a significant role in tumor progression. Moreover, although the better OS and PFS for recurrent CCs operated with a resective aim in comparison to those treated with a palliative goal corresponds to routine clinical observations, a possible selection bias can affect this result.

## 9 Conclusions

EEA can represent an effective approach to treat loco-regional recurrent CCs, since its characteristics of minimal invasiveness, low morbidity rate, straight and direct access with limited brain and neurovascular structures manipulation. It is well tolerated, with a satisfactory preservation of patients QoL and a low rate of post-operative neurological deficits. Conversely, it results effective to achieve resolution of symptoms, as diplopia, trigeminal nevralgia, facial and orbital pain and nasal obstruction.

At the same time, EEA in these patients is very challenging and should be performed in dedicated centers. The achievement of surgical goal is largely depending on surgeon experience and appropriate technological equipment, which are of paramount importance to avoid potentially disastrous complications, favored by the distortions of the normal anatomy and the presence of scars or the increase in tumor fibrousness due to the previous treatments.

For such challenges, a strict selection of surgical indications is necessary. Particularly, EEA can be considered for those patients with consistent perspectives of GTR and further adjuvant therapies, or for selected cases that, even if in poorer conditions and with limited possibilities of further radiation or chemotherapies, have significant chances of symptoms alleviation and at least temporarily QoL restoring by surgery. Future prospective studies involving larger cohorts of patients are strongly required to better identify predictors of outcome and, thus, improving tailored patient care.

## Disclosures

The authors declare that the content of this manuscript has not been published before, in part or in full, and has not been submitted elsewhere for review.

The authors also declare to have no conflict of interest to disclose.

No financial support was received to support the study nor the generation of this manuscript.

## Declaration of competing interest

The authors declare that they have no known competing financial interests or personal relationships that could have appeared to influence the work reported in this paper.
